# The Healing Effect of Bioglue in Articular Cartilage Defect of Femoral Condyle in Experimental Rabbit Model

**DOI:** 10.5812/kowsar.20741804.2254

**Published:** 2011-09-15

**Authors:** N Tanideh, S Dehghani Nazhvani, F Mojtahed Jaberi, D Mehrabani, S Rezazadeh, S Pakbaz, A Tamadon, B Nikahval

**Affiliations:** 1 Stem Cell and Transgenic Technology Research Center, Department of Pharmacology, Shiraz University of Medical Sciences, Shiraz, Iran; 2 Department of Veterinary Surgery, School of Veterinary Medicine, Shiraz University, Shiraz, Iran; 3 Stem Cell and Transgenic Technology Research Center, Department of Orthopedic Surgery, Shiraz, Iran; 4 Stem Cell and Transgenic Technology Research Center, Department of Pathology, Shiraz, Iran; 5 Division of Animal Health Management, School of Veterinary Medicine, Shiraz University, Shiraz, Iran

**Keywords:** Healing, Bioglue, Articular Cartilage, Defect, Femoral Condyle, Rabbit

## Abstract

**Background:**

The full-thickness articular cartilage defects of knee have a poor healing capacity that may progress to osteoarthritis and need a knee replacement. This study determines the healing effect of bioglue in fullthickness articular cartilage defect of femoral condyle in rabbit.

**Methods:**

Forty-eight male rabbits were randomly divided into four equal groups. In group A, 4 mm articular cartilage defects were created in the right and left medial femoral condyles. Then a graft from xiphoid cartilage was transferred into the defect together with a designed bioglue and the knees were closed. In group B, an articular cartilage defect was created identical to group A, but the defect size was 6 mm. In group C, 4 and 6 mm articular cartilage defects were created in the right and left medial femoral condyles respectively. The graft was transferred into the defect and the knees were stitched. In group D, articular cartilage defects were created similar to group C, just filled with bioglue and closed. The rabbits were euthanized and subgroups were defined as A1, B1, C1 and D1 after 30 days and A2, B2, C2 and D2 after 60 days. The cartilages were macroscopically and histologically investigated for any changes.

**Results:**

Microscopic and macroscopic investigations showed that bioglue had a significant healing effect in the femoral condyle.

**Conclusion:**

Addition of bioglue can effectively promote the healing of articular cartilage defects.

## Introduction

Articular hyaline cartilage has an important role in distribution of mechanical loads on the joints.[1] It is an avascular tissue and has a poor healing potential after partial thickness defects.[2][3] The lesions do not reach the subchondral bone and do not have any access to the progenitor cells of the bone marrow. Platelet-rich plasma (PRP) has several growth factors stimulating the healing process. PRP can treat tendonitis, muscle injuries, ligament sprains or tears and bursitis, but more researches on PRP are necessary based on orthopedic problems such as cartilage defects, osteoarthritis, bone healing, etc.[3]

Cartilage defects may be associated with immobility, pain, stiffness, decreased quality of life, and can potentially result into severe osteoarthritis in longterm. [4][5] Cartilage defects are most commonly seen in the knee joint mostly due to trauma.6 Many cartilage repair procedures are introduced as open surgical procedures (osteotomy and distraction of joints), cartilage transplantation, tissue engineering, autologous chondrocyte implantation, autologous condition plasma, pridie drilling and microfracture, perichondral and periosteal grafts, fetal membranes, and intra-articular hyaluronan injection.[5][6][7][8][9][10][11][12][13][14][15][16] There are several methods of fixation of autologous cartilage graft including polydioxanone pins, krishner wires, Herbert screws, cyanoacrylate adhesive, polymethylmethacrylate cement, stainless steel nails, and suture.[17][18][19] The aim of this study was to evaluate the healing effect of bioglue in experimentally-induced femoral condyle articular cartilage defect in rabbit as an animal model.

## Materials and Methods

A 2 ml blood sample was provided from each rabbit and transferred into a 3.2% trisodium citrate container to prepare rabbit plasma. Thromboplastin-D was used to perform the one-stage prothrombin time (PT). The reagent composition was <0.9% rabbit brain tissue, 0.08% sodium azide, 2% buffer, salts and stabilizers.

The designed trephine was a stainless autoclavable steel instrument with the length of the base of 9 cm and the handle of 11 cm. There was a hole at the end of base where a rod was inserted for transformation of the graft cartilage. At the end of the trephine’s handle, there was a hole with a diameter of 6 mm and the other trephine’s handle had a diameter of 4 mm.

Forty-eight male rabbits were randomly divided into four equal groups. In group A, 4 mm articular cartilage defects were created in the right and left medial femoral condyles leaving the subchondral bone intact with a 4 cm median parapattellar approach. Both medial femoral condyles were exposed using a trephine. The incision on the xiphoid cartilage was 3 cm and trephined it with a 4 mm length. The graft was transferred into the medial femoral condyle with a designed bioglue. The bioglue included two components of surgical adhesive containing rabbit plasma (2 drops) and thromboplastin-D reagent (one drop) filled in the defect. Then both knees were closed by sutures. Retinaculum layers were sutured by 3.0 vicryl in a continuous pattern and skin by nylon 2.0 in an interrupted pattern. Both feet were immobilized by bandage until 12 h after surgery and then positioned in passive motion.

In group B, an articular cartilage defect was created identical to group A, but the defect size was 6 mm. In group C, 4 and 6 mm articular cartilage defects were created in the right and left medial femoral condyles respectively. Then a graft was provided from xiphoid cartilage and transferred into the defect and the knees were stitched. In group D, 4 and 6 mm articular cartilage defects were created in the right and left medial femoral condyles respectively. Then the defects were filled with bioglue and were closed by sutures. The rabbits were euthanized and subgrouped as A1, B1, C1, and D1 after 30 days and A2, B2, C2, and D2 after 60 days. The cartilages were macroscopically and histologically investigated for any changes. Modified International Cartilage Repair Society (ICRS) visual histological assessment scale (Table 1) and histological grading scale for the defects of cartilage (Table 2) were used for histological studies applying H and E, toluidine blue, and safranin O staining methods.

**Table 1:** International Cartilage Repair Society (ICRS) visual histological assessment scale.

**Features**	**Scores**
Surface	
Smooth/Continuous	3
Discontinuities/Irregularities	0
Matrix	
Hyaline	3
Mixture: Hyaline/Fibrocartilage	2
Fibrocartilage	1
Fibrous tissue	0
Cell distribution	
Columnar	3
Mixed/Columnar-clusters	2
Clusters	1
Individual cells/Disorganized	0
Cell population viability	
Predominantly viable	2
Partially viable	1
< 10% viable	0
Subchondral bone	
Normal	3
Increased remodeling	2
Bone necrosis/Granulation tissue	1
Detached/Fracture/Callus at base	0
Cartilage mineralization (calcified cartilage)	
Normal	1
Abnormal/Inappropriate location	0
Type I collagen staining of the matrix	
Normal or nearly normal	3
Moderate staining	2
Slight staining	1
None	0
Type II collagen staining of the matrix	
Normal or nearly normal	3
Moderate staining	2
Slight staining	1
None	0

**Table 2:** Histological grading scale for the defects of cartilage.

**Categories**	**Points**
Cell morphology	
Hyaline cartilage	0
Mostly hyaline cartilage	1
Mostly fibrocartilage	2
Mostly non-cartilage	3
Non-cartilage	4
Matrix-staining (metachromasia)	
Normal (compared with host adjacent cartilage)	0
Slightly reduced	1
Markedly reduced	2
No metachromatic stain	3
Surface regularity	
Smooth (> 3/4)	0
Moderate (> 1/2-3/4)	1
Irregular (1/4-1/2)	2
Severely irregular (< 1/4)	3
Thickness of cartilage	
> 2/3	0
1/3-2/3	1
< 1/3	2
Integration of donor with host adjacent cartilage	
Both edges integrated	0
One edge integrated	1
Neither edge integrated	2

All the experiments were carried out under aseptic conditions in the Laboratory Animal Center of Shiraz University of Medical Sciences and care and the sacrifice procedure all adhered to the guidelines and was under supervision of Animal Care Committee of Iran Veterinary Organization. The study was approved in the University Ethics Committee. All animals underwent anesthesia during the study using ketamin (44 mg/kg) and xylazine (5 mg/kg).

SPSS software (Version 15, Chicago, IL, USA) was used for statistical analysis using a nonparametric Mann-Whitney U test. The data, presented as median, range, and mean on graphs which designed by GraphPad Prism software (version 5.01, USA). P<0.05 was statistically considered significant.

## Results

**Fig. 1:**
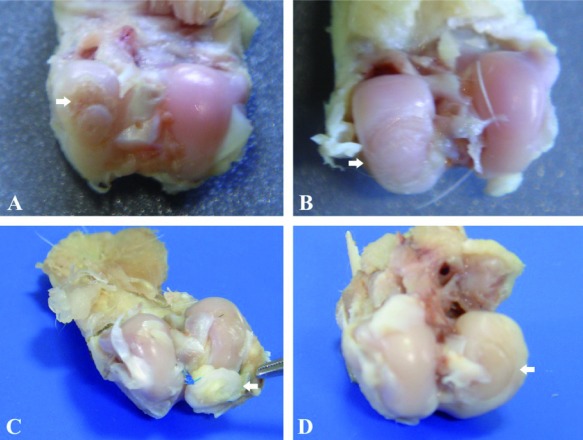
Grossly evaluation of repair articular cartilage of medial femoral condyle in different groups of male rabbits (arrows). A, cartilage graft fixed with designed BioGlue (size 4 mm, time 60 days); B, cartilage graft fixed with designed BioGlue (size 6 mm, 30 days); C, cartilage graft fixed with suture (size 4 mm, time 30 days); D, designed BioGlue filled cartilage defect (size 4 mm, time 30 days).

**Fig. 2:**
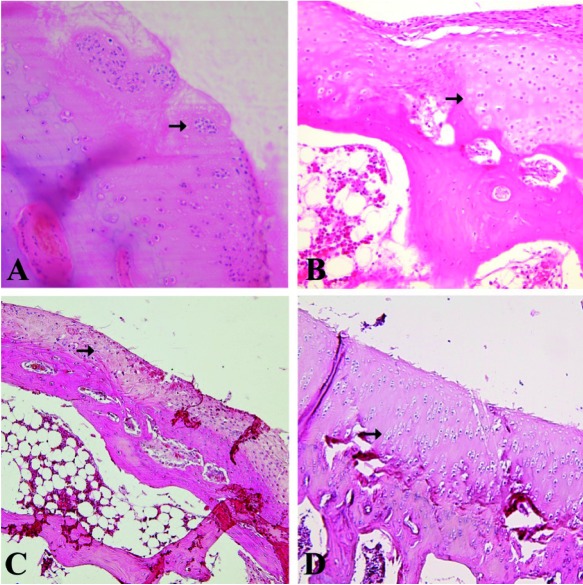
High-power photomicrographs of medial femoral condyle of male rabbit obtained on day 60 with H and E that shows histological changes in relation to healing effects between all groups. A) A2 group, microscopic appearance of reparative tissue showing groups (clusters) of chondrocytes in an extracellular matrix (arrow). The surface is irregular and subchondral area showed granulation tissue formation and remodeling (original magnification×40). B) B2 group, microscopic appearance of reparative tissue showing mixed hyaline/fibrocartilaginous character (arrow). Cartilaginous part revealed predominantly viable individual cells. Tidemark was irregular and interrupted (original magnification×20). C) C2R group, microscopic appearance of reparative tissue showing fibrocartilaginous and fibrous character (arrow). Cartilaginous parts revealed individual disorganized and partially viable cells. (original magnification ×20); D) D2L group, microscopic appearance of reparative tissue showing relatively smooth repaired hyaline cartilage containing columnar arrangement of chondrocytes (arrow; original magnification ×20).

**Fig. 3:**
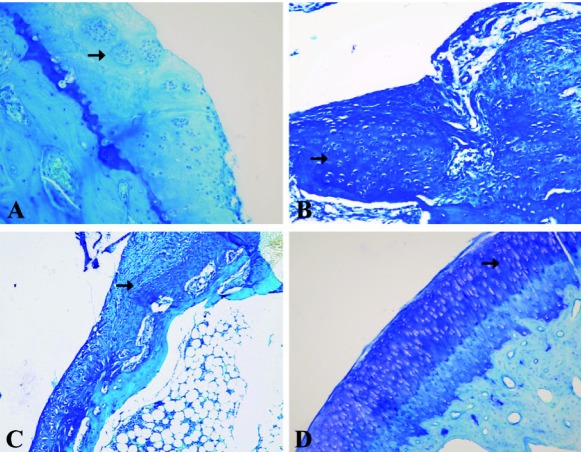
High-power photomicrographs of medial femoral condyle obtained on day 60 with toluidene blue that shows histological changes in relation to healing effects between all groups. A) A2 group, histochemical staining of toluidene blue showing defect repaired with hyaline-like cartilage. The subchondral bone and tidemark were well remodeled (original magnification ×40). B) B2 group, histochemical staining of toluidene blue showing densely stained reparative tissue and irregular tidemark (original magnification ×40). C) C2R group, histochemical staining of toluidene blue showing absence of a well formed tidemark (original magnification ×20). D) D2L group, histochemical staining of toluidene blue showing nearly normal cartilage staining. The surface was smooth and the tidemark was well remodeled (original magnification ×40).

**Fig. 4:**
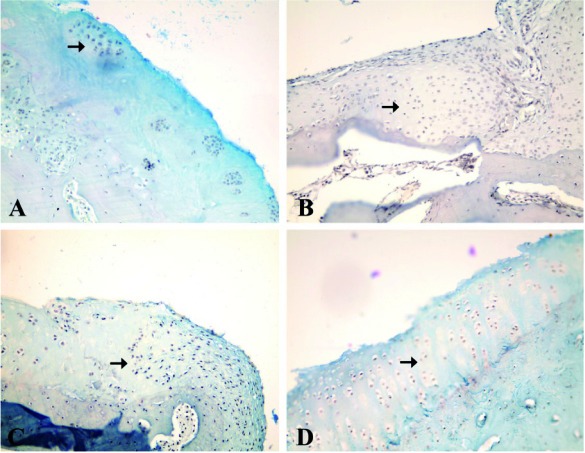
High-power photomicrographs of medial femoral condyle obtained on 60 days with safranin O that shows histological changes in relation to healing effects between all groups (safranin O ×40). A) A2 group, histochemical staining of safranin O that was more intense in the superficial layer (original magnification×40). B) B2 group, histochemical staining of safranin O with minimal matrix staining (original magnification×40). C) C2R group, histochemical staining of safranin O showing slight staining of extracellular matrix in fibrocartilaginous parts (original magnification ×40). D) D2L group, histochemical staining of safranin O showing moderate staining of extracellular matrix, more intense in the superficial layer (original magnification ×40).

Figure 1 shows macroscopic changes in articular surfaces in different groups. In group D, the surface of articular cartilage was more smooth and continuous than other groups followed by group A. Figure 2 shows H and E histological changes in relation to healing effects between all groups. Figure 3 demonstrates changes for toluidene blue histological changes in relation to healing effects between all groups. Figure 4 denotes to safranin O histological changes in relation to healing effects between all groups.

**Fig. 5:**
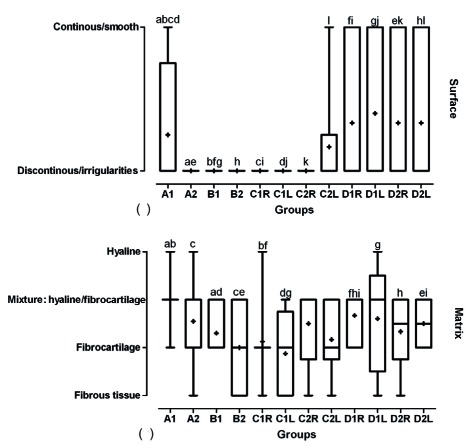
Box and whisker plot of median, mean, and range of changes in surface (a) and matrix (b) of cartilage based on modified ICRS visual histological assessment to evaluate designed BioGlue on articular cartilage graft in male rabbits. A, designed BioGlue and cartilage graft (4 mm); B, designed BioGlue and cartilage graft (6 mm); C, cartilage graft sutured; D, cartilage defect filled with BioGlue; 1, followed after 30 days; 2, followed after 60 days; R, right knee; L, Left knee. The same superscript letters indicates significant statistical difference between comparable groups using Mann-Whitney U test.

**Fig. 6:**
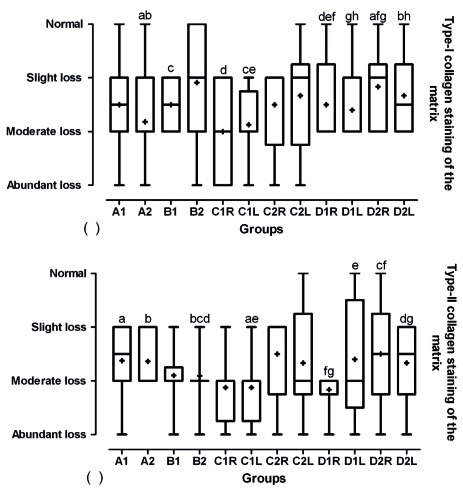
Box and whisker plot of median, mean, and range of changes in type I collagen staining of the matrix (a) and type II collagen staining of the matrix (b) of cartilage based on modified ICRS visual histological assessment to evaluate designed BioGlue on articular cartilage graft in male rabbits. A, designed BioGlue and cartilage graft (4 mm); B, designed BioGlue and cartilage graft (6 mm); C, cartilage graft sutured; D, cartilage defect filled with BioGlue; 1, followed after 30 days; 2, followed after 60 days; R, right knee; L, Left knee. The same superscript letters indicates significant statistical difference between comparable groups using Mann-Whitney U test.

According to ICRS visual histological assessment scale, four indices of articular cartilage evaluation presented the effects of different treatment as followed. Evaluation of cartilage surface showed that in group D, the surface was smooth and continuous in shape and the healing was significantly more prominent in comparison to other groups (p<0.05, Figure 5a). There were significant differences between grades of cartilage matrix of different groups and the group D had more mixture of hyaline and fibrocartilage (p<0.05, Figure 5b). The grades of the type-I collagen staining of the matrix based on ICRS table indicated that group D as compared with the other groups had more normal amount of type-I collagen (p<0.05, Figure 6aa). Regarding ICRS in relation to type II collagen staining of the matrix variable, indicated that group D as compared with the other groups have significantly more normal in amount of type-II collagen (p<0.05, Figure 6b).

**Fig. 7:**
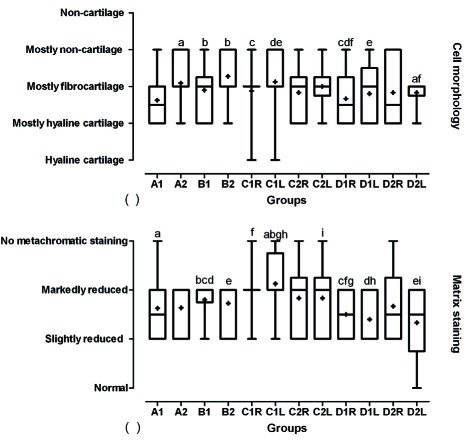
Box and whisker plot of median, mean, and range of changes in cell morphology (a) and matrix staining (b) based on histological grading scale to evaluate designed BioGlue on articular cartilage graft in male rabbits. A, designed BioGlue and cartilage graft (4 mm); B, designed BioGlue and cartilage graft (6 mm); C, cartilage graft sutured; D, cartilage defect filled with BioGlue ; 1, followed after 30 days; 2, followed after 60 days; R, right knee; L, Left knee. The same superscript letters indicates significant statistical difference between comparable groups using Mann-Whitney U test.

Regarding table of histological grading scale for the defects of cartilage, only two indices of articular cartilage evaluation presented the effects of different treatment as followed. There were significant differences between cell morphology of different groups and group D as compared with the other groups had more hyaline cartilage (p<0.05, Figure 7a). Moreover, there were significant differences between matrix staining of different groups and group D as compared with the other groups that were more normal in staining of matrix (p<0.05, Figure 7b).

## Discussion

It was shown that bone-marrow mesenchymal stem cells have chondral and osteogenic potential in joint microfractures.[20] Full-thickness defects of the weightbearing regions of the articular cartilage are repaired with hyaline-like cartilage after implantation of autologous osteochondral progenitor cells isolated from bone-marrow or periosteal tissue and grown in cell culture.[20][21][22] Transforming growth factor-beta (TGF-β) stimulates synthesis of proteoglycans that are present in rabbit plasma.[23]

In this study in group C, a delay in tissue repair was noticed due to the presence of the suture as a foreign body in the region.9 Articular cartilage defects smaller than 4 mm in diameter have only limited intrinsic capacity for self-repair that were identical to our findings.[24]

Designed bioglue had rich growth factors that stimulate healing response in a more power full form3 were observed in our study too. However, after 60 days, formation of type II collagen was more than subjects after 30 days. It showed that designed bioglue in small amount was more effective than a large volume.

In an ultrastructural study of articular-thickness defects in rabbits, Fuller and Ghadially[25] confirmed the fact that articular cartilage failed to generate a significant repair reaction capable of filling the defect. However, we showed some repair. In the surviving cartilage, the graft showed evidences of high metabolic activity and remodeling of the surface of the defect which was covered by a layer of new matrix containing fine collagen fibers deployed tangentially to the articular surface.[26]

Studies of articular cartilage defects in animals with the use of pins, Krishner wire, fibrin sealant, sutureless, polymethylacrylate, cell different origin, periosteum costal perichondrial graft, hyaluronic acid, and allogeneic chondrocytes showed conflicting results. The use of designed bioglue and autologous cartilage graft also minimized the likelihood transmitting infectious disease and rejection.[27]

The current investigation demonstrated repair of articular cartilage in group D that cartilage defect was filled with the designed bioglue. Designed bioglue consisted of thromboplastin-D and rabbit plasma that this mixture was rich in growth factors that stimulated proliferation of chondrocytes as one of the possible explanation for group D that was better than other groups. The designed bioglue did not have enough adhesion for fixation of cartilage graft that was harvested from xiphoid cartilage. Therefore, the graft in groups A and B did not have enough connection to the site of operation resulting in the graft to be as a loose body in joint interaction with cartilage repair. If we used bandage as longer, it seems that we would get good results in groups A and B. The procedures to elicit repair that were described in this report may lead to a clinically useful method of treatment for defects of the articular cartilage.

Our findings were similar to Amirghofran et al.,[28] and Rakei et al.,[29] which showed that bioglue as available, safe, and cost-effective glue, can be used in all operations without any complication. In conclusion, this procedure had considerable relevance to the treatment of defects in the cartilage of humans and provides the basis for the development of a repair technology that is capable of regenerating large areas of articular cartilage.
